# Supplementary material for: An osmolality/salinity-responsive enhancer 1 (OSRE1) in intron 1 promotes salinity induction of tilapia glutamine synthetase
Source: Sci Rep. 2020 Jul 21;10:12103. doi: 10.1038/s41598-020-69090-z (PMC7374092; doi:10.1038/s41598-020-69090-z)
Supplement: Supplementary file 1 — Supplementary Information. [file 41598_2020_69090_MOESM1_ESM.pdf]

Supplementary Information

An osmolality/ salinity-responsive enhancer 1 (OSRE1) in intron 1 promotes salinity induction of tilapia glutamine synthetase

Chanhee Kim[1] and Dietmar Kültz[1]*

[1]Biochemical Evolution Laboratory, Department of Animal Sciences, University of California, Davis, CA, 95616

*dkueltz@ucdavis.edu

Supplementary Figure S1. Additional targeted SWATH-MS/Skyline protein quantitation data of GS protein in cells grown in four different medium conditions (Dosing: isosmotic (315 mOsmol/kg), hyperosmotic (650 mOsmol/kg), isosmotic+ 10µM actinomycin D, hyperosmotic+10µM actinomycin D). Two different peptides of glutamine synthetase are shown in panels **A)** EEGEEPANYSK and **B)** RPSANCDPYAVTEALVR with five biological replicates for each dosing condition.

Figure S1A

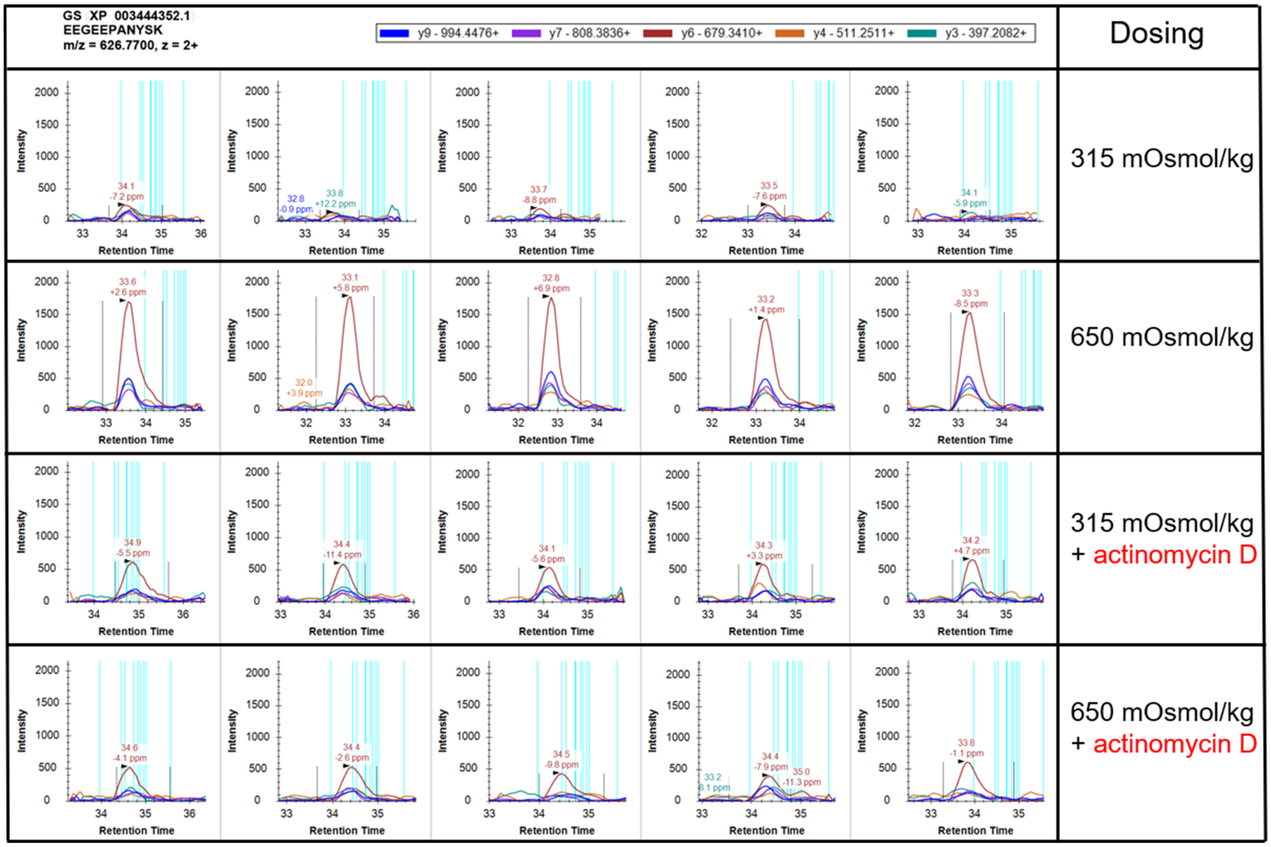

Figure S1B

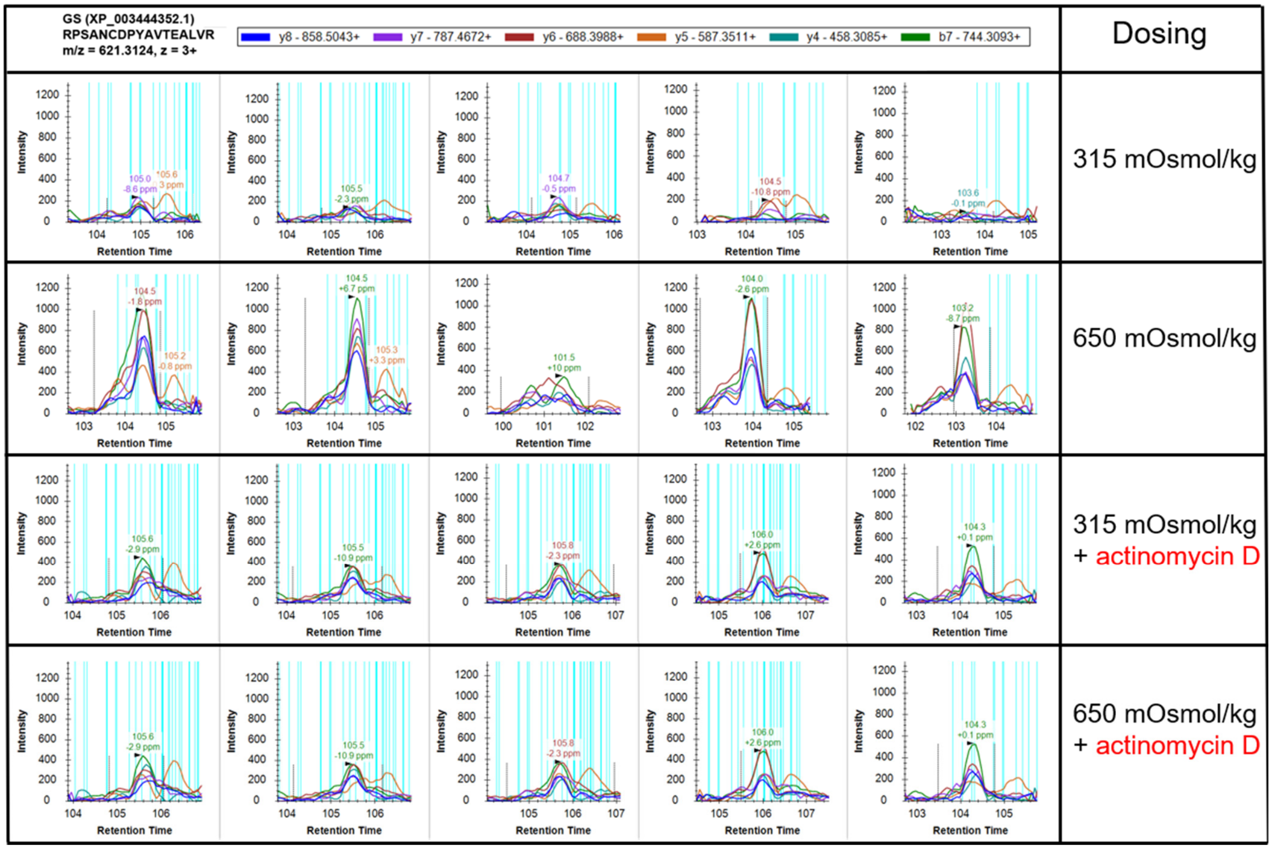
GS (XP_003444352.1)
RPSANCDPYAVTEALVR
m/z = 621.3124, z = 3+
y8 - 858.5043+
y7 - 787.4672+
y6 - 688.3988+
y5 - 587.3511+
y4 - 458.3085+
b7 - 744.3093+
Dosing
315 mOsmol/kg
650 mOsmol/kg
315 mOsmol/kg + actinomycin D
650 mOsmol/kg + actinomycin D
Intensity
Retention Time

Supplementary Figure S2. Plasmid map of the initial luciferase reporter construct consisting of pGL4.23 vector (blue) and an insert representing the 3.4-kb 5' regulatory sequence (RS) of ***GS*** (orange, -2825 to +499). The 3' end of the insert (+499) represents the start codon (SC). Exon 1, a truncated Exon 2 (dark grey), intron 1 (green) of ***GS*** and the luciferase reporter gene (yellow) are indicated. The vector map image was generated with Geneious 11.0 (Biomatters, https://www.geneious.com).

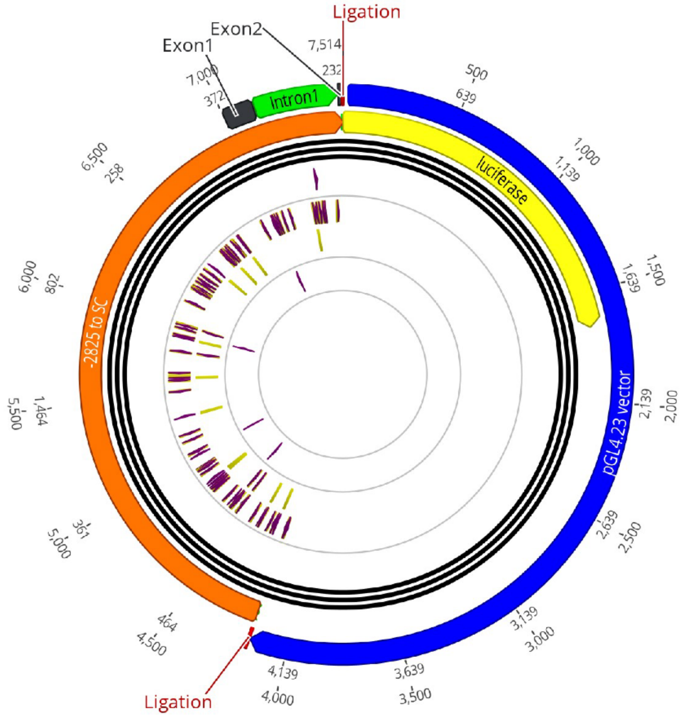

Supplementary Figure S3. The representative sequencing result from the reporter constructs showing a successful selective deletion of ***GS***-OSRE1 from intron 1. The alignment image was generated with Geneious 11.0 (Biomatters, https://www.geneious.com).

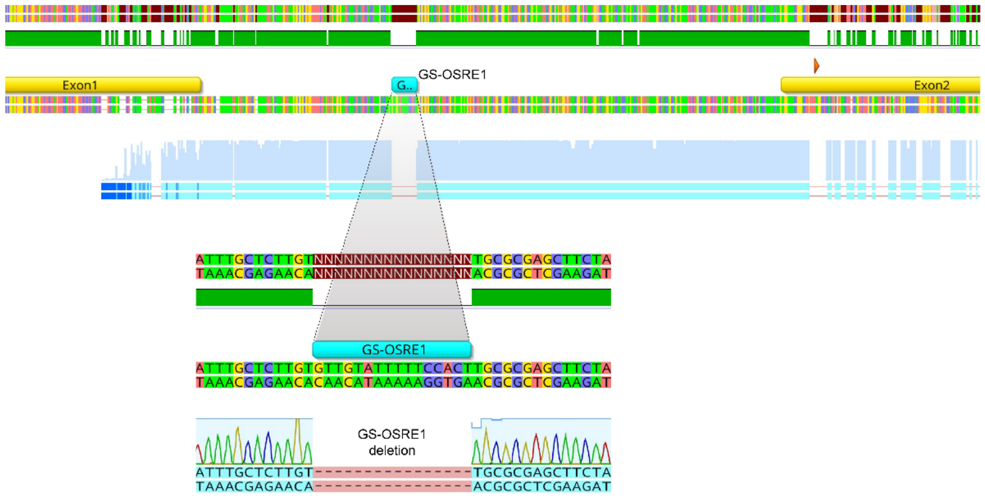

Supplementary Table S1. Synthetic oligonucleotide sequences containing different number of copies of ***GS***-OSRE1 (SacI and HindIII restriction sites also included) and the corresponding Forward & Reverse primers sequences for their amplification. Red and black color variation separates multiple ***GS***-OSRE1 copies.

GS-OSRE1: GTTGTATTTTTCCACT

Number of copy	Whole oligonucleotide sequence (bp length)	Forward primer sequence	Reverse primer sequence
**1**	CCCCCGAGCTCCATGCATGCATGCTGTGTTGTATTTTTCCACTTGCATGC<br>ATGCATGCCGAGCTAAGCTTGGGGG (75 bp)	CCCCCGAGCTCCATGCATGCATG<br>CTGTGTTGTATTTTTCCACTTGCA<br>TGCATG	CCCCCAAGCTTAGCTCGGCATG<br>CATGCATGC AAGTGGAA
**2**	CCCCCGAGCTCCGTTGTATTTTTCCACTACTGGTTGTATTTTTCCACTCGA<br>GCTAAGCTTGGGGG (65 bp)	CCCCCGAGCTCCGTTGTATTTTT<br>CCACTACTGGTTGTATTTTTCC	CCCCCAAGCTTAGCTCGAGTGG<br>AAAAATACA ACCAGTAGTGG
**3**	CCCCCGAGCTCTGTGTTGTATTTTTCCACTTGCACTGTGTGTTGTATTTTT<br>CCACTTGCACTGTGTGTTGTATTTTTCCACTTGCAAGCTTGGGGG (96 bp)	CCCCCGAGCTCTGTGTTGTATTT<br>TTCCACTTGCACTGTGTGTTGTAT<br>TTTTCCAC	CCCCCAAGCTTGCAAGTGGAAA<br>AATACAACCACAGTGCAAGTGG<br>AAAAATACAACACAC
**4**	CCCCCGAGCTCTGTGTTGTATTTTTCCACTTGCACTGTGTGTTGTATTTTT<br>CCACTTGCCGAGCTTGTGTTGTATTTTTCCACTTGCACTGTGTGTTGTATT<br>TTTCCACTTGCAAGCTTGGGGG (124 bp)	Genestrands synthesis (Eurofins Genomics)	
**5**	CCCCCGAGCTCTGTGTTGTATTTTTCCACTTGCACTGTGTGTTGTATTTTT<br>CCACTTGCACTGTGTGTTGTATTTTTCCACTTGCACTGTGTGTTGTATTTTT<br>CCACTTGCACTGTGTGTTGTATTTTTCCACTTGCAAGCTTGGGGG (148 bp)	Genestrands synthesis (Eurofins Genomics)	
